# Benign mixed Müllerian (duct) vaginal tumor in a 12-y-old goat

**DOI:** 10.1177/10406387211069370

**Published:** 2022-01-10

**Authors:** Svenja Hartung, Elfi K. Schlohsarczyk, Alexandra Jost, Marlene Sickinger, Kernt Köhler

**Affiliations:** Institute for Veterinary Pathology, Faculty of Veterinary Medicine, Justus-Liebig-University Giessen, Giessen, Germany; Institute for Veterinary Pathology, Faculty of Veterinary Medicine, Justus-Liebig-University Giessen, Giessen, Germany; Clinic for Ruminants, Internal Medicine and Surgery, Faculty of Veterinary Medicine, Justus-Liebig-University Giessen, Giessen, Germany; Clinic for Ruminants, Internal Medicine and Surgery, Faculty of Veterinary Medicine, Justus-Liebig-University Giessen, Giessen, Germany; Institute for Veterinary Pathology, Faculty of Veterinary Medicine, Justus-Liebig-University Giessen, Giessen, Germany

**Keywords:** goats, mixed Müllerian tumor, neoplasia, spindle-cell epithelioma

## Abstract

In human and veterinary medicine, mixed Müllerian tumors (MMTs) are rarely diagnosed neoplasms of the tubular female genital tract. Although there are case reports of malignant MMTs in various species, benign MMTs have only been described once in a macaque. Here we present a case of benign MMT in a 12-y-old goat, and review the literature on uterine, cervical, and vaginal neoplasia in goats. The doe was presented with vaginal discharge and was euthanized because of the high suspicion of intraabdominal neoplasia. On gross examination, an ulcerated vaginal mass was identified. Histologically, 2 distinct cell populations were present: smooth muscle cells that were well differentiated and positive for alpha–smooth muscle actin, and ciliated columnar epithelial cells that lined ductal structures and had no signs of malignancy. These findings led to the diagnosis of neoplasia of Müllerian origin. Benign MMT should be considered as a differential diagnosis for uterine and vaginal neoplasms in goats.

Neoplasms of the tubular female genital tract are rare in goats. Throughout domestic species, uterine adenocarcinomas, leiomyomas, and leiomyosarcomas are the most commonly diagnosed tumors.^7,10^ The overall tumor prevalence in goats is relatively high at 8.7%; the neoplasm described most frequently is lymphoma.^11^ Benign mixed Müllerian tumor (benign MMT), also known as spindle-cell epithelioma, has not been described previously in companion or livestock species, including ruminants, to our knowledge. An atypical polypoid adenomyoma of the oviduct (benign MMT) has been reported in a cynomolgus macaque (syn. crab-eating macaque; *Macaca fascicularis*).^16^ Benign MMT is characterized by a benign spindle-cell population containing ductal epithelial cells and myoepithelial cells, the origin of which is still controversial.^8,12^ The epithelial structures are postulated to originate from the urogenital sinus or Müllerian duct.^12^ Pluripotent progenitor cells and remnants of vestibular glands on the hymenal ring have also been discussed as potential tissues of origin.^8,18^ Vaginal MMTs without myoepithelial differentiation have also been described.^1,2^ The malignant form, also known as uterine carcinosarcoma, is also rare in both humans and animals. Uterine malignant MMT has been described in a 13-y-old goat with anorexia and lethargy.^4^

A 12-y-old female goat (*Capra aegagrus hircus*) had a 3-wk history of white, mucoid vaginal discharge, and had reduced general condition and feed intake. The animal had previously been treated with meloxicam and unidentified antibiotics. Clinical examination of the goat revealed an adequate nutritional score, reduced intestinal peristalsis, a body temperature of 39.1°C, and matted coat in the perianal area as a result of vaginal discharge. Ultrasonographic examination revealed a 10.5-cm diameter mass in the caudal abdominal cavity. Clear assignment to a specific organ was not possible at that time. The urinary bladder was not clearly identifiable. Hematologic examination and serum chemistry revealed moderate leukocytosis (21.0 × 10^9^/L; RI: 4.0–7.4 × 10^9^/L), mild hypokalemia (3.1 mmol/L; RI: 3.8–5.5 mmol/L), and mild hyperglycemia (5.7 mmol/L; RI: 2.1–3.7 mmol/L). The owner declined surgery because of the animal’s advanced age, and the doe was euthanized because of its poor general condition and the suspicion of urinary or uterine neoplasia.

On gross examination, a 10 × 15 × 25-cm, pedunculated, round-to-oval, soft white mass was found in the vaginal lumen (Fig. 1A). The mass had a thin 1 × 1 × 3-cm pedicle connected to the cranioventral vaginal wall near the cervix. The surface of the mass was erythematous, ulcerated, and covered with white mucinous exudate. There was no evidence of metastasis or other remarkable pathologic findings. Samples of the mass were fixed in 10% neutral-buffered formalin. The samples were processed routinely, and sections were stained with H&E, Goldner, and Van Gieson stains. Immunohistochemistry was performed to detect alpha–smooth muscle actin (αSMA; Dako), vimentin (Dako), desmin (Dako), pan-cytokeratin (OriGene), and Ki67 (Dako) as a proliferation marker. A biotinylated horse anti-mouse antibody (Vector) was used as a secondary antibody.

**Figure 1.**
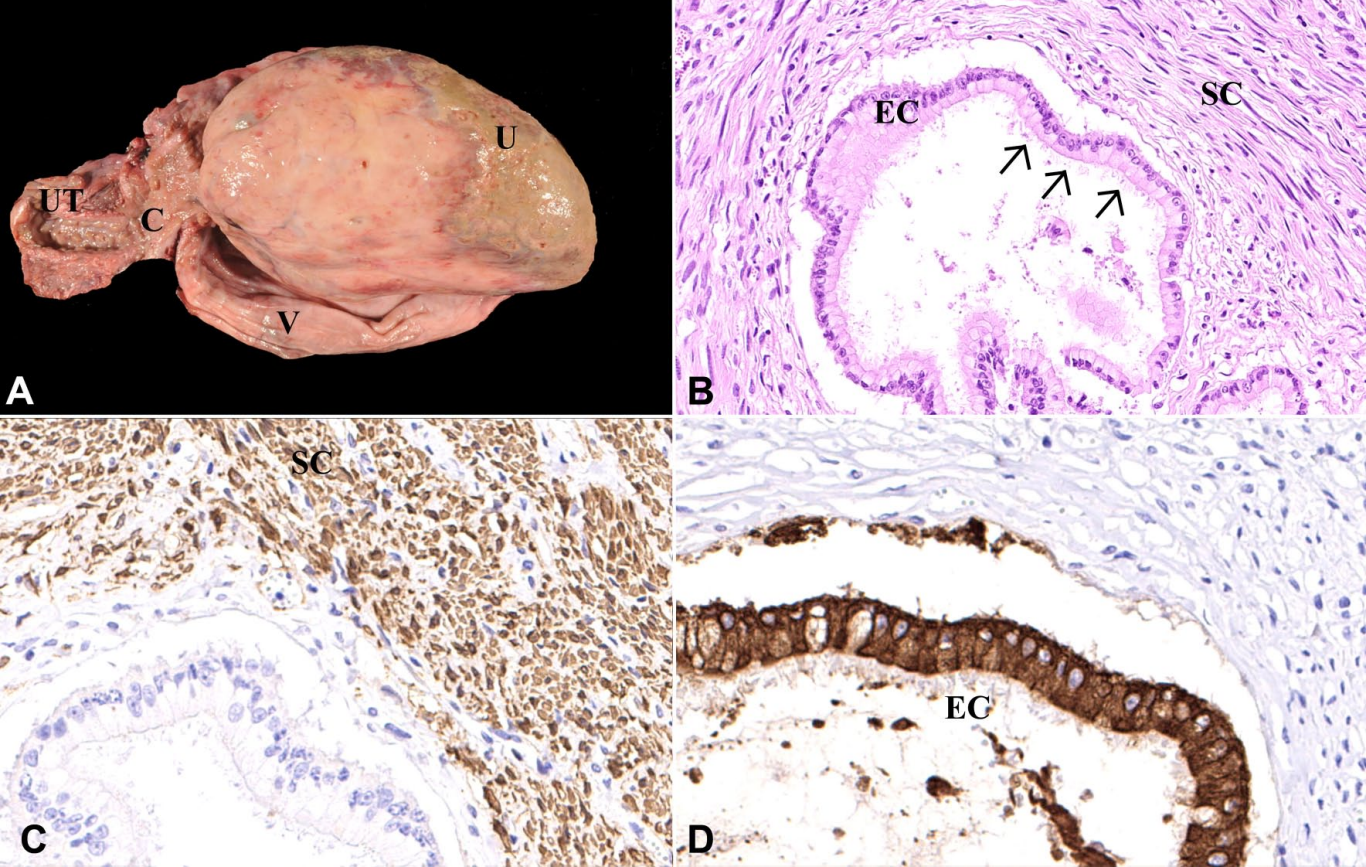
Benign mixed Müllerian tumor in the vagina of a goat. **A.** A 10 × 15 × 25-cm mass in the vaginal lumen (V) with ulceration (U); cervix (C) and uterus (UT) without pathologic findings. **B.** The tumor consists of 2 cell populations: a well-differentiated spindle-cell (SC) population and ductal structures lined by a mostly columnar ciliated epithelium (EC). Cilia are marked by arrows. H&E. 20×. **C.** Immunohistochemistry for smooth muscle actin; the spindle-cell population (SC) stains brown (= positive). 20×. **D.** Immunohistochemistry for pan-cytokeratin; the columnar ciliated epithelial cells (EC) stain brown (= positive). 20×.

Histopathology revealed a densely cellular, thinly encapsulated mass containing 2 distinct cell populations. The mass consisted of a large number of well-differentiated spindle cells (staining red in the van Gieson stain, and dark-green to brown in the Goldner stain) with oval-to-elongated nuclei containing finely stippled chromatin and only one nucleolus per nucleus (Fig. 1B). The cytoplasm was brightly eosinophilic. The cells were arranged in wave-like strands and bundles surrounded by a moderate number of epithelial canaliculi. The epithelial cells had pale eosinophilic cytoplasm and round-to-oval nuclei with finely stippled chromatin (Fig. 1B). In addition, both cell populations showed no signs of malignancy, including a mitotic count of <1 in 2.37 mm^2^, and only mild anisokaryosis and anisocytosis. Immunohistologically, the spindle-cell population was positive for αSMA (Fig. 1C) and negative for vimentin, desmin, and pan-cytokeratin, confirming smooth muscle origin. The ductal ciliated epithelium was positive for pan-cytokeratin (Fig. 1D) and negative for αSMA, vimentin, and desmin. With the proliferation marker (Ki67), there were up to 4 positive nuclei per hpf in the spindle-cell population and up to 2 positive nuclei per hpf in the ciliated epithelium. Based on the appearance of 2 different cell populations, a benign MMT was diagnosed.

In goats, there are few case reports of neoplasms of the tubular female genital tract (Table 1). In most cases, older goats tend to be affected, and leiomyoma, leiomyosarcoma, and adenocarcinoma of the uterus appear to be diagnosed most commonly. Malignant MMT has been reported in animal species, including dogs, rats, mice, cats, and rabbits,^4^ and is characterized by epithelial and mesenchymal components and signs of malignancy. Vaginal neoplasms are only rarely reported in goats. Uterine adenocarcinomas are often ulcerated and have a cauliflower-like appearance. Histologic examination reveals tubular and acinar structures surrounded by vascularized stroma. Necrosis and infiltrative growth are common. Uterine adenocarcinomas are similarly rare in other animal species, with the exception of rabbits, cattle, cats, and some strains of rats.^17^ Immunohistologic examination is a useful tool for differentiation of neoplasms of the tubular female genital tract, and, in small ruminants especially, for the reliable diagnosis of uncommon tumor variants, such as benign MMT.

**Table 1.** Reported cases of female tubular genital tract neoplasia in goats.

Reference	Animal	Location	Diagnosis
^10^	42 goats of different breeds	Uterus, vagina, cervix	13 leiomyomas, 13 adenocarcinomas, 11 leiomyosarcomas, 2 sarcomas, 1 anaplastic carcinoma, 1 fibrosarcoma, 1 adenoma
^11^	Goats of diverse breeds	Uterus	1 cervical and uterine adenocarcinoma, 2 uterine leiomyomas
		Cervix	Cervical adenocarcinoma
		Vagina	Adenocarcinoma, leiomyoma
		Vulva	Squamous cell carcinoma
^20^	Aged Saanen goat	Uterus, cervix	Leiomyosarcoma
^5^	Aged mixed-breed goat	Uterus	Collision tumor (adenocarcinoma and leiomyosarcoma)
^9^	11-y-old goat	Uterus	Endometrial adenocarcinoma
^13^	Dwarf goat	Uterus	Leiomyoma
^15^	7-y-old Saanen goat	Uterus	Leiomyosarcoma
^3^	Goat	Cervix	Leiomyoma
^4^	13-y-old mixed-breed goat	Cervix	Uterine carcinosarcoma (malignant MMT)
^14^	Goat	Cervix	Leiomyoma
^19^	Aged Toggenburg goat	Cervix	Leiomyoma
^6^	Mature Saanen-type goat	Vagina	Leiomyofibromatosis

MMT = mixed Müllerian tumor.
